# Identifying the Origin of Hexagonal Boron Nitride Single Photon Emitters with nano-FTIR

**DOI:** 10.1021/acs.jpclett.5c03632

**Published:** 2026-02-11

**Authors:** Chia-Hung Wu, Po-Sheng Shih, Nicholaus Kevin Tanjaya, Kuo-Ping Chen, Satoshi Ishii

**Affiliations:** ∇ College of Photonics, National Yang Ming Chiao Tung University, 301 Gaofa 3rd Road, Tainan 71150, Taiwan; ‡ Research Center for Materials Nanoarchitectonics (MANA), National Institute for Materials Science (NIMS), 1-1 Namiki, Tsukuba, Ibaraki 305-0044, Japan; § Institute of Photonics Technologies, 34881 National Tsing Hua University, Hsinchu 300, Taiwan; ∥ Subprogram in Materials Science and Engineering, Graduate School of Science and Technology, University of Tsukuba, Tsukuba, Ibaraki 305-8577, Japan

## Abstract

Quantum light sources in van der Waals solid systems operating at room temperature have drawn significant attention, among which, with particular interest, is hexagonal boron nitride (hBN). Numerous efforts have focused on producing reliable, bright, and controllable single photon emitters (SPEs) in hBN. However, the identity of these emitters remains ambiguous, leading to unreproducible experimental results. Here, we offer direct evidence that hBN SPEs generated through annealing are not inside the hBN itself but originate from organic residues that carbonize and form aromatic fluorophores. Nanoscale Fourier transform infrared spectroscopy is used to analyze the emission sites, revealing the presence of carbon bonds in aromatic rings with a characteristic C=C absorption peak at 1650 cm^–1^. These emitters are primarily located in encapsulated areas of the hBN flake rather than uniformly distributed within the lattice. This finding opens the door to designing stable and reliable SPEs, enabling their integration into the rapidly growing quantum photonics applications.

## Graphical abstract

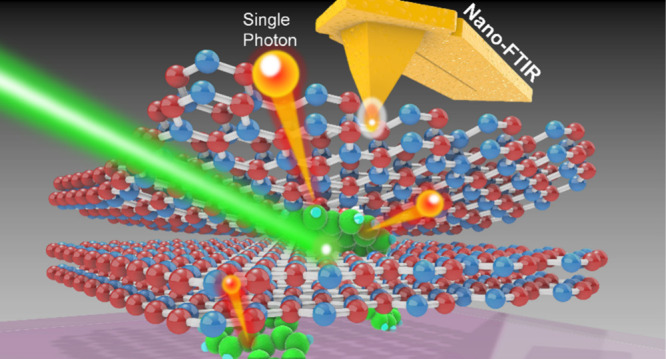

Quantum computing is currently the solution to next-generation communication technology. In recent years, attention has been drawn to the study of quantum photonic chips realized on solid-state platforms. The antibunching effect of photons introduced by single photon emitters (SPEs) is the key requirement to many quantum information applications, such as metrology,1 networking, and computing.^1–5^ In terms of SPEs, they have been discovered in molecules,^6–8^ quantum dots,^9,10^ diamonds^3,11,12^ and large band gap van der Waals materials such as transition-metal dichalcogenides (TMDCs)^13–15^ and hexagonal boron nitride (hBN).^1,8,16–20^ The generation of SPEs in molecules and quantum dots requires cryogenic and high vacuum environments for their emission purity and chemical instability. Diamonds produce stable emissions at room temperature due to NV centers from the defect sites. However, they are costly and difficult to fabricate.^21^ TMDCs need nanostructures that may increase local density of states (Purcell effect),^15^ or create a defect state in the energy band structure induced by lattice strain.^14,22^

Hexagonal boron nitride has been reported to host single photon emissions at room temperature, receiving intense attention as a promising candidate for quantum information applications. van der Waals materials are also a compatible platform for standard lithographic processes, making them desirable for future integration with photonic integrated circuits. Many have reported bright and stable emission of SPEs in hBN at the visible range (1.6 to 2.5 eV).^23^ Various methods have been proposed to obtain these SPEs in hBN platforms, such as ion beam irradiation,^24–26^ electron beam irradiation,^27^ strain induced defect state, and annealing.^28–30^ The debate over the origin of hBN SPEs has arisen from the diversity of observed sample-preparation methods and emission types. Earlier studies suggested that carbon-based defects are likely responsible for the emission near 2 eV,^19,20,31–34^ which are the most commonly observed class of hBN SPEs. N. Mendelson et al. controlled the incorporation of carbon impurities in the hBN lattice via ion implantation with a TEM grid as a mask, followed by annealing, where they observed region-controlled stable single-photon emission in the visible range at room temperature.^19^ M. Neumann et al. reported that aromatic fluorophores generated from organic residues are the origin of hBN SPEs.^8^ They argued that hBN acted as an encapsulation layer, preventing the photon emitters from being photobleached, where similar SPEs were also observed by replacing hBN with mica. Their work was reproduced by others.^35,36^ These works indicate that emission originates from contaminants rather than from vacancy defects in the lattice. However, most studies on this topic relied on far-field optical measurements, photoluminescence (PL) spectroscopy, photon lifetime, and photon correlation, all of which provided indirect information about the emitters. Material analysis methods to characterize chemical bonds, like energy-dispersive X-ray spectroscopy (EDX), Raman spectroscopy, and Fourier transform infrared spectroscopy (FTIR) are unsuitable for this issue due to the nanoscale size of the SPE specimens.

To study the addressed issue, the emission sites were scanned by the near-field scanning probe Fourier transform infrared spectroscopy (nano-FTIR) to identify the chemical bonding composition in molecules. We elucidated that the presence of emission was strongly correlated with carbon bonds (C=C) in aromatic rings, which exhibit a characteristic peak at 1650 cm^–1^. By studying the phase spectra at higher harmonic orders, we propose that the emitters in hBN are primarily buried at the interface between the substrate and the flake. This statement is based on the variation in probing depth at different demodulation orders *n* (typically *n* > 1), where a higher order provides a smaller probing volume, thus revealing the 3D structure of the sample.^37,38^ We emphasize that this manuscript addresses the most widely reported hBN SPE emission type, which occurs close to 2 eV. Other emission types are beyond the scope of this work. The knowledge of the origins of hBN SPEs enables more reproducible and scalable designs for future implementations of these quantum signal sources.

To clarify the ongoing debate of visible range hBN SPE origin, we approach the verification of a hypothesis that indicates organic molecules are the cause of hBN emission.^8^ Therefore, two types of samples were fabricated in this study, which were labeled Type I and Type II throughout the manuscript. These samples will be described in detail in the following text. Figure 1(a–f) shows the step-by-step process of Type I samples. Mechanically hBN exfoliated with a polydimethylsiloxane (PDMS) sheet thicknesses ranging from a few to hundreds of nanometers were stamped onto the substrate, as depicted in Figure 1(b). Organic residues and contaminants from the previous steps were removed by oxygen cleaning in a furnace at 700 °C and atmospheric pressure for 3 h. After cooling down, the sample was immersed in anisole for another 30 min and annealed in nitrogen at 850 °C for 1 h (Figure 1(d and e)). Under oxygen-free, high-temperature conditions, anisole underwent condensation reactions, forming polycyclic aromatic hydrocarbons (PAHs). PAHs are large and planar molecules composed of fused benzene rings, many of which belong to the family of aromatic fluorophores known for their fluorescence properties.^39,40^ For Type II samples, the oxygen annealing step was omitted to preserve the contaminants, which kept the dangling bonds on the substrate and hBN surface.^41^

**Figure 1:**
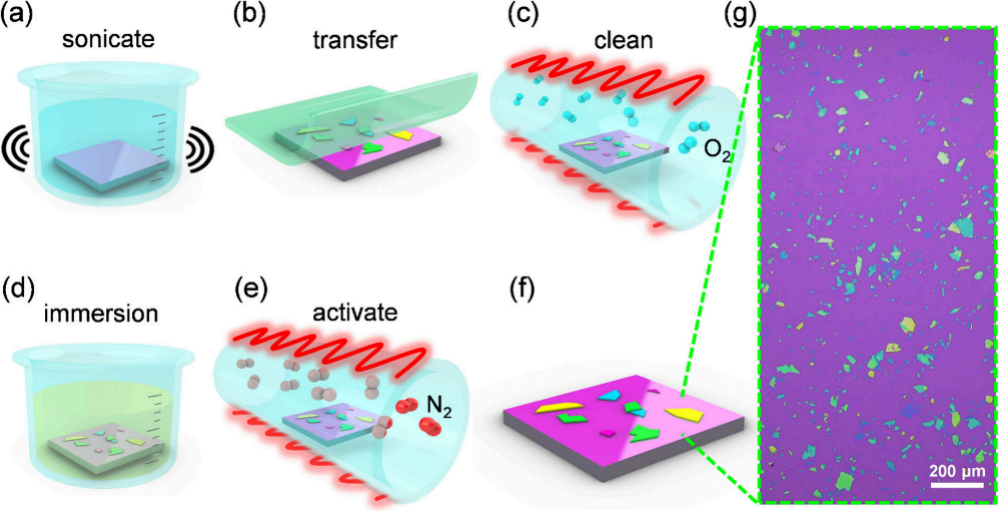
Schematic of the sample process procedures. (a) Sonication cleaning of substrates in acetone and IPA for 10 min in sequence. (b) Mechanical exfoliation of hBN flakes from bulk crystal with PDMS, then transferred to the substrate. (c) Cleaning in oxygen at a high temperature to remove organic residues from the previous steps. (d) Sample immersion in anisole for 30 min to control the introduction of organic contaminants into the system. (e) Generation of emitters by annealing in an inert atmosphere at 850 °C for 1 h. (f) Schematic and (g) optical microscope image of the sample.

To begin with, we compare the luminescence and nano-FTIR spectra on a Type I specimen. Figure 2(a) shows a composite image of atomic force microscopy (AFM) image and PL mapping of an hBN flake (Flake 1). As shown in Figure S1, the AFM image indicates that Flake 1 is a combination of two hBN flakes stacked together caused by overlapping during the mechanical exfoliation process. Interestingly, emission sites are mostly limited to the overlapped areas, as shown in Figure 2(a). This is due to the formation of “mini-gaps” caused by contaminants trapped between Van Der Waals layers from the exfoliation transfer step.^41^ With oxygen heat treatment, contaminants were mostly removed from the hBN/substrate interface, introducing stronger adhesion. Even after this treatment, some contaminants and mechanically formed gaps still remained in the stacked areas, though reduced. These mini-gaps further allowed organic solvents to diffuse between the stacks and aggregate in the following fabrication procedures (Figures 1(d) and 1(e)), thereby creating sandwiched photon emitters. The spectra of with (point A), without (point C) PL emission, and graphite G-band (point B) were shown in Figure 2(c). A 610 nm emission peak was observed at point A, dwarfing out the D, G and 2D-bands of graphite at 573, 583, and 630 nm, respectively.^42^ Emissions around 610 nm were also observed for other emitters, as shown in Figure S2. Here, the observation of antibunching in photon correlation measurement with a g^(2)^(0) < 0.5 at Point A shows the presence of single photon emission as depicted in Figure 2(e). At point B, apart from the hBN Raman signal at 573 nm, which was excited with a 532 nm laser, we also observed the existence of graphite D, G and 2D bands. Point C was extracted for reference in a pure hBN area. In addition, the blinking nature of the emitters is presented in Figure S3. The results in Figure 2(d) demonstrate polarization-dependent characteristics of an SPE, where the PL intensity varies with the excitation laser polarization angle, exhibiting dipole-like behavior consistent with previous reports.^20,43,44^ Furthermore, PLE analysis, detailed in Figure S4, was conducted on the luminescent sites without an antibunching effect, as shown in Figure S5, showing polarization-independent behavior. In Figure S6, we also measure the polarizations of three other SPEs, all exhibiting dipole-like behavior with distinct polarization angles. This implies that the luminescent sites were composed of aggregated emitters and were unrelated to the hexagonal lattice of the hBN flake.^45^

**Figure 2:**
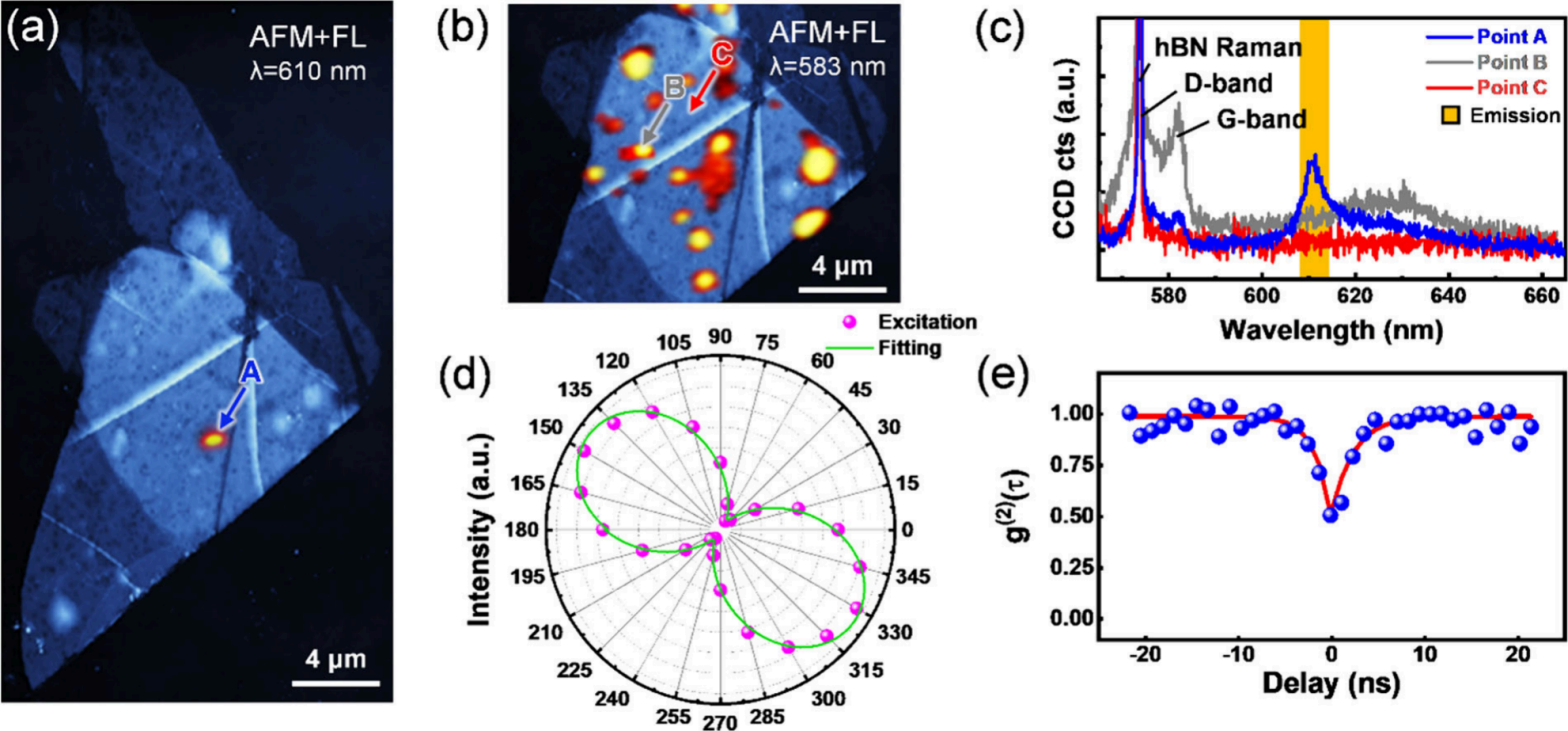
(a) Composite image of AFM and FL mapping for Flake 1, a blue arrow is added for clarity, presenting sites with 610 nm emission. (b) Composite image of AFM and carbon G-band mapping for Flake 1, gray and red arrows are added for clarity, presenting sites with and without graphite Raman signals. (c) Spectra obtained with 532 nm laser excitation showcasing hBN, graphite Raman characteristic peaks and emission center at 610 nm. (d) PL intensity as a function of excitation laser polarization angle at point A. The green curve is the fitted sine curve of the measured data. (e) Photon correlation measurement at point A. The g^(2)^(0) < 0.5 shows single photon emission behavior of this luminescent site. The blue dots represent experimental data, whereas the red line represents the fitted curve.

To gain deeper insight into the interaction between the emitter and its environment, IR scattering and AFM images were acquired using a commercial nano-FTIR system (see Figure S7) as shown in Figure 3(a) and (b), respectively. To simplify the discussion, Flake 1 was divided into three regions labeled I, II, and III as depicted in Figure 3(a). In region I, dark spots or patches were visible in contrast to the “red” background on the flake, indicating suppression of scattering signals. Compared with the AFM image in Figure 3(b), these dark patches were small holes and were most likely caused by etching during the oxygen heat treatment, revealing the SiO_2_ substrate below. In region II, dark patches still exist. However, the surface morphology indicates that not all “red” patches are small holes but bumps that are formed while immersion in the anisole step. Organic solvents infiltrate the mini-gaps between the flakes, aggregating to form bubbles visible in AFM scans. During the inert atmosphere heat treatment process, these solvents transformed into polycyclic aromatic compounds, some of which were graphite patches and others were aromatic fluorophores. This transformation, being a random process, explains the wide variety of color centers observed in the previous literature.^23,43^ The suppression of scattering signals in the IR spectrum at bubble areas is due to the high carbon and hydrogen content, which promotes absorption and lowers scattering due to limited probing depths compared to pristine hBN. To clarify the nature of emission sites, the IR spectra measured at point A were examined. Figure 3(d) shows the third-order amplitude values, where both points show the hBN signature peak at 1370 cm^–1^ without a significant difference. On the other hand, as shown in Figure 3(e) bottom panel, the C=C vibrational mode at 1650 cm^–1^ was observable at point A but absent in point C for the third-order phase spectra (φ_3_). The phase spectra qualitatively resemble absorption spectra in organic molecular systems.^46^ Figure S8 shows that the same C=C vibrational mode at 1650 cm^–1^ is also observed in different locations. The broad absorption peak ranging from 1350 cm^–1^ to 1600 cm^–1^ is the signature of the hBN itself, as its appearance was consistent in all measurements, and the peak width was related to flake thickness (see Supporting Information Figure S9). Given the emission wavelength of 610 nm in hBN, it is probable that the emitting species were aromatic fluorophores comprising multiple conjugated aromatic benzene rings. A single benzene ring, with its large bandgap, usually emits in the ultraviolet range.^47,48^ With the emitters being identified as polycyclic aromatic compounds, the emitters’ distribution was traced by a line scan that passed through point A with the nano-FTIR, where the scanning points were 50 nm apart. In Figure 3(f), positions exhibiting the C=C peak at 1650 cm^–1^ were labeled as blue stars for better visibility. From the scan results, photoluminescent areas exhibit higher counts of C=C signals, indicating a clear correlation between the two. Furthermore, the distribution of the labeled signals shown in Figure 3 (f) clarifies why the second-order intensity correlation function measurement is typically taken at the edge of the emission site.

**Figure 3:**
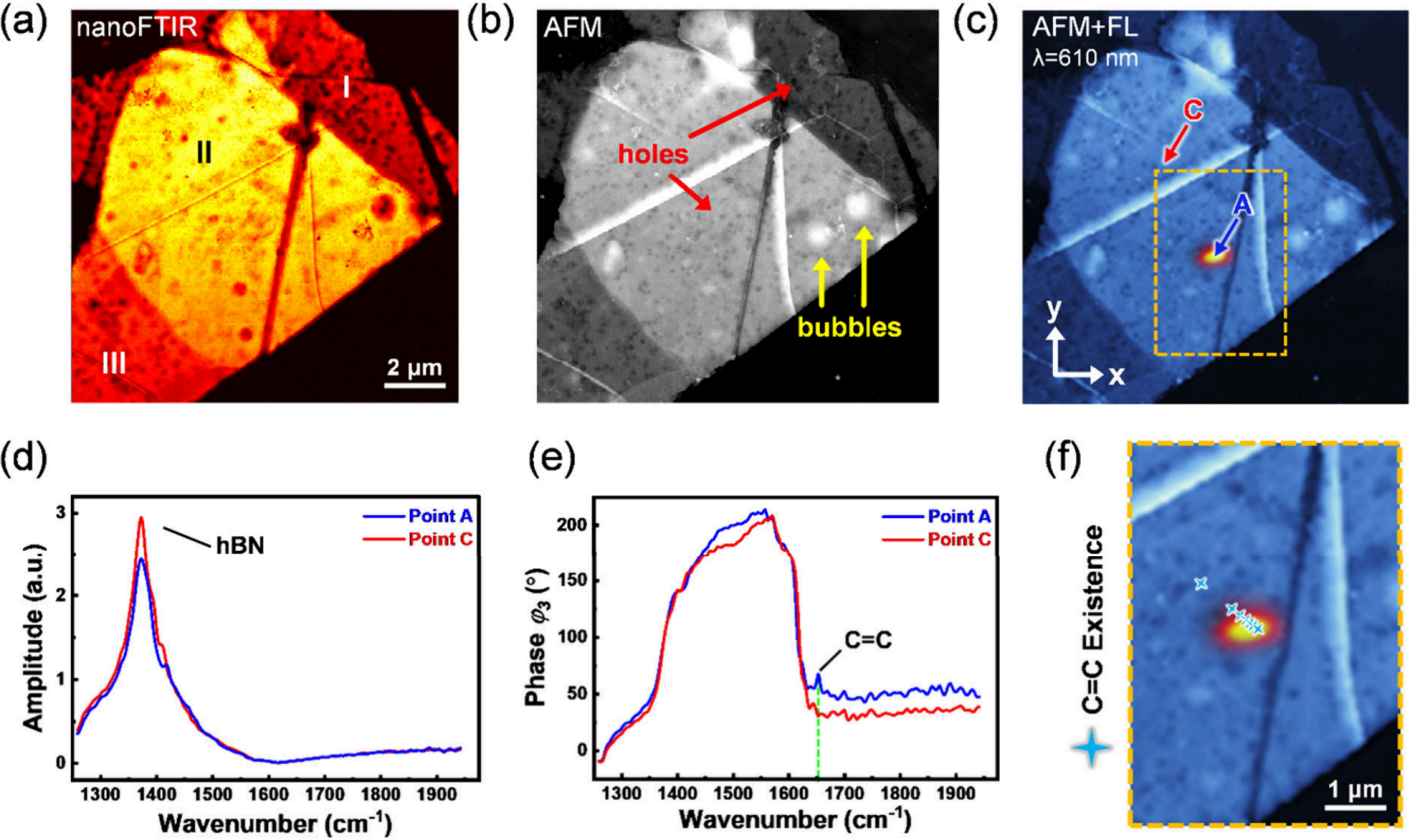
Comparison of results obtained from near and far fields inspection. (a) Tip detected IR scattering image from Flake 1. Different regions of the flake are labeled as I, II and III. Region II is the overlapped area of two hBN flakes. (b) AFM topography of Flake 1, enabling visualization of bubbles, wrinkles and holes. (c) PL and AFM composition plot which is identical to Figure 2(a). (d) Amplitude spectrum obtained from nanoFTIR at points A and C as depicted in (c). (e) 3rd order phase spectrum obtained from nanoFTIR at points A and C, where point A has an emission center at 610 nm as shown in Figure 2(d). (f) 1650 cm^–1^ signal existence taken by a line scan near point A, which is a single photon emitter.

Information on the locations of fluorescent substances along the thickness direction is vital for identifying the emitter origin and for future applications. The probing depth in nano-FTIR depends on the demodulation order, as higher harmonic signals provide shallower penetration depths.^38,49^ This method enables the study of buried nanostructures or components and facilitates 3D sample reconstruction. Here, Type II hBN specimens with thicknesses of 6, 15, 20, 30, 50, and 100 nm were investigated as shown in Figure 4(a–f). By omitting the oxygen cleaning step in the Type I process, the dangling bonds of surface contaminants were preserved, forming a uniform layer of graphite and possibly PAHs on the sample surface after immersion in anisole and high-temperature oxygen-free treatment, as shown in Supporting Information Figure S10 and Figure 5(d), respectively. The generated graphite layer on the hBN surface was removed with the 532 nm CW laser exposure for 5 min until the signal of carbon G-band stabilized. This step is to visualize fluid motion through the presence of carbonized organics and mimic Type I specimens, as there were no carbon substances on top of the hBN flake. Photobleaching phenomena will be explained in detail in Figure 5. Specimen thickness profiles were measured with AFM as depicted in the lower panels of Figure 4(a–f). Figure 4(g) depicts the third-order phase spectrum of nano-FTIR, where the most substantial C=C signal at 1650 cm^–1^ came from the 20 and 30 nm thick flakes, characterized by a distinct sharp peak. The 50 and 15 nm flakes demonstrated similar signal intensities, which were notably smaller than those of the 20 and 30 nm flakes. The signal was absent in the 100 nm flakes, and a small peak was still present in the 6 nm flakes. The explanation for these observations is as follows: Fixing the demodulation order determines the probing depth of the measurement. Flakes with thicknesses ranging from 20 to 30 nm offer the highest signal for φ_3_^*C*=*C*^. The decrease in signal height observed in Figure 4(g) for specimens thicker than the optimal range, like the 50 and 100 nm flakes, indicates that the fluorescence substances are beyond the probing depth and that the hBN emitters are buried between the substrate and the flake. This could potentially lead to reduced signal strength as the signal intensity of subsurface features increases compared to surface ones.^50^ Conversely, flakes thinner than the optimal thickness face photobleaching issues due to inadequate emitter protection. The phase spectra for demodulation orders n = 2 and n = 3 for a 100 nm thick hBN flake are compared in Figure 4(h). An observable peak at 1650 cm^–1^ was present in the φ_2_ spectra but absent in the φ_3_ spectra. This is because the dipole’s penetration depth at the tip apex was deeper at lower harmonic signal demodulation orders. Therefore, it is confirmed that the hBN photon emitters were sealed underneath the surface, encapsulated at the interface between the flake and the substrate. Additionally, the photon lifetimes of these emitters across different flake thicknesses were measured and are provided in Table S1 of the Supporting Information.

**Figure 4:**
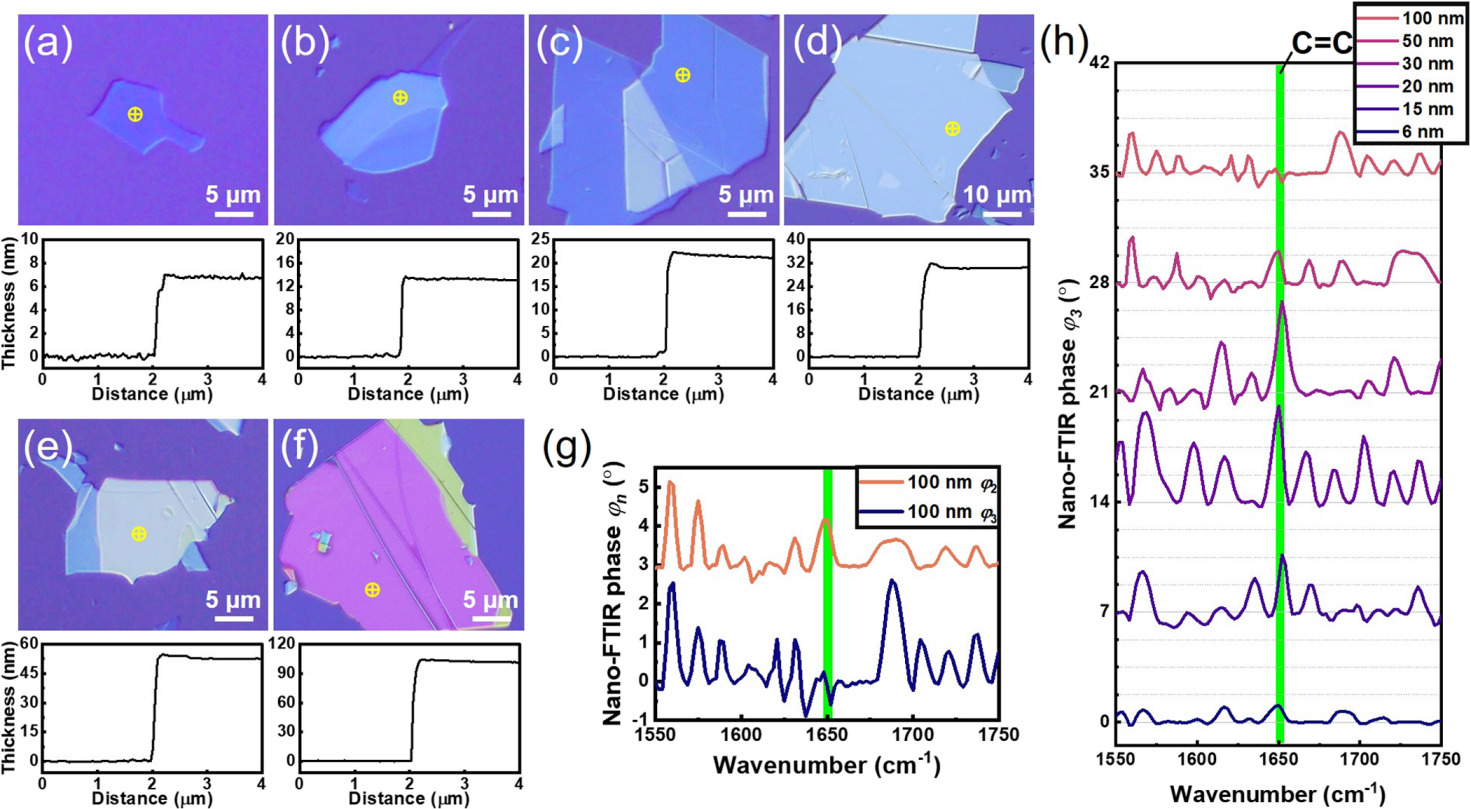
(a–f) Optical microscope image and AFM thickness profile of Type II specimen with different hBN thicknesses, ranging from 6 to 100 nm. (g) 3rd demodulation order of phase spectra obtained from different thickness specimens. (h) Phase spectra of 3rd and 2nd order harmonics obtained from the 100 nm thick hBN flake. The green shade is added to aid the eyes, indicative of the 1650 cm^–1^ signal.

**Figure 5:**
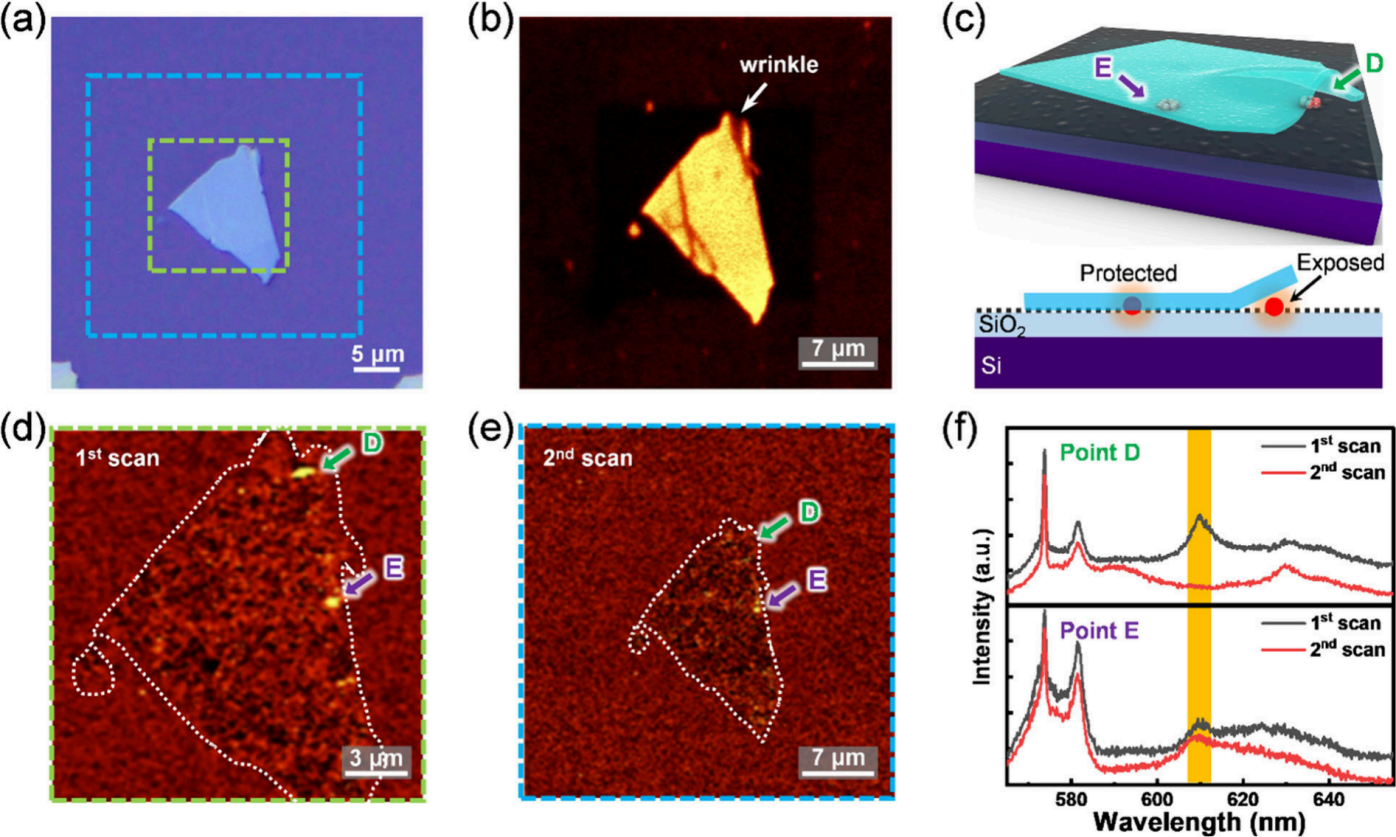
Demonstration of photobleaching PAH emitters on hBN. (a) Optical microscope image of a Type II specimen. The green and cyan dashed boxes illustrate the areas of the 1st and 2nd laser scanning regions, respectively. (b) Raman mapping image of the 2nd scan, indicating the area of the hBN flake. (c) Schematic of the investigated specimen with two emitter sites, where one is encapsulated (point E) and the other is exposed to ambient atmosphere (point D). The black dashed line in the bottom panel indicates the presence of a graphite patch. (d and e) 610 nm emission mapping of 1st and 2nd laser scanning, respectively. Dashed white line is added to indicate the boundary of the hBN flake. (f) Luminescence spectrum extracted from 1st and 2nd scans on the hBN at points D and E with 532 nm CW laser excitation.

Photobleaching of hBN SPEs have been reported in previous literature. This phenomenon was primarily caused by inadequate protection, which led to the modification of defect sites upon oxygen exposure under irradiation.^17^ Here, we present a clear visualization of photobleaching phenomena, indicating that emission color centers in the visible range do not originate from local hBN lattice defect states. Figure 5(a) displays a bright field image of a Type II hBN specimen captured using an optical microscope (BX53, OLYMPUS). Figure 5(b) presents the Raman map of the 1370 cm^–1^ peak, clearly outlining the location and extent of the hBN flake. A comparison of white light and Raman signals reveals surface wrinkles, attributed to differences in the focal planes of the excitation laser and white light imaging. This contrast suggests that emission site E is encapsulated by hBN, whereas site D remains exposed to ambient conditions, as illustrated in Figure 5(c). The green and blue dashed boxes in Figure 5(a) denote the regions scanned during the first and second laser passes, respectively. Figures 5(d) and (e) display the 610 nm photoluminescence maps, revealing the presence and photobleaching of emitters across the specimen. In addition, the 582 nm emission map reveals widespread graphite residue beyond the hBN domains, as shown in Supporting Information Figure S10. This is consistent with the expected contamination due to the absence of post-transfer oxygen annealing following the PDMS stamping process. Returning to the discussion of the 610 nm emitters, Figure 5(f) shows that following the first laser scan (i.e., during the second scan), emission from site D is no longer observed, while site E retains its photoluminescence. This outcome aligns with our expectations based on the different environmental exposures of the two sites. Graphite signals were also detected due to the laser beam spot size (∼3 μm) exceeding the scale of individual PAH molecules. Analysis of the fluorescence intensity of the D, G, and 2D bands indicates a reduction in signal postirradiation, although the graphite structure remains largely intact. This contrasts with the complete degradation of PAHs, underscoring their greater sensitivity to laser excitation. To assess the protective role of the hBN flake, time-resolved photoluminescence under continuous 532 nm excitation was monitored (Supporting Information Figure S10). After 90 s of illumination, the Raman signal of graphite stabilized. Notably, graphite encapsulated with hBN retained ∼ 25% of its signal, while unprotected graphite was entirely bleached.

This study provides compelling experimental evidence that links hBN emission centers in vicinity to 2 eV to the carbon signal of aromatic rings (C=C) at 1650 cm^–1^. By carefully analyzing luminescent mapping and nano-FTIR results, our findings contrast with previous studies suggesting that hBN lattice defects caused single photon emission. Our conclusion is supported by comprehensive investigations of photophysical properties, including comparison of emitters under different hBN protection conditions and location of emitters. Nano-FTIR phase spectra let us pinpoint emitter locations with nanoscale resolution. The emitters form aggregations from fluid motion at the mini-gaps, where high-temperature processing turns organic residues possibly into graphite or molecular fluorophores with benzene rings responsible for the emissions. We found a higher emitter density in emission centers. Additionally, analysis of harmonic orders showed that these emitters were encapsulated and protected beneath the van der Waals structure. Although the lattice defect was ruled out for emission around 2 eV, we continue to promote hBN as a crucial material for quantum light source applications, considering its advantageous properties, such as its atomically thin structure, large band gap, and its inert encapsulating layer that shields single photon emitters from photobleaching in oxygen-rich conditions. With a deeper understanding of how hBN SPEs are generated, we believe this work can accelerate the development of hBN-based quantum light sources by offering a fresh perspective that leads to more rational designs and controllable results. It could also be beneficial for the reproducible and scalable design of future nanophotonics devices integration, such as the bullseye gratings,^51^ bound states in the continuum (BIC) metasurfaces,^52^ and silicon photonics.^32^

## Experimental Section/Methods

### Device Fabrication

Type I and II samples were fabricated starting from a 300 nm silicon dioxide deposition by an electron beam evaporation system (VT1-10CE, ULVAC) on a silicon wafer. After the deposition, the substrate was precleaned by sonication in acetone and isopropanol for 10 min each subsequently. Then, mechanically exfoliated hBN was transferred onto the substrate, followed by a cleaning process to enhance hBN adhesion and eliminate organic contaminants from previous steps. The cleaning process was performed by annealing in the ambient air at 700 °C for 3 h. Note that the cleaning process was omitted for Type II samples to preserve the contaminants for lower hBN adhesion to the substrate. Then the sample was immersed in anisole (Cas No. 100-66-3, 99%, FUJIFILM Wako Pure Chemical Corporation) for 30 min to introduce controlled organic contaminants. To generate emitters, the samples were subjected to 850 °C annealing for 1 h in an inert atmosphere (99.99% N_2,_ 1 atm). All the annealing steps were processed in a tube furnace with a temperature gradient of 2 °C/second.

### hBN Exfoliation

High-quality commercial hBN crystals from hq+ graphene were used as the starting material. PDMS film (PF-40 × 40-0170-X4, Gel-Pak) was utilized for exfoliation. The flakes were obtained through repeated exfoliation, with two PDMS strips being stuck together and peeled apart about 20 times. After this process, the hBN flakes were transferred to the target substrate by adhering the PDMS strip to the substrate and peeling it off, leaving the flakes on the surface.

### Photoluminescence Measurement

The PL spectra of the hBN emitters were obtained on a WITec alpha 150 microscope using a 100× objective (MPLFLN, NA = 0.9, OLYMPUS) and a 532 nm CW laser. The PL areal scans were acquired with an incident laser intensity of 5 mW and an exposure time of 500 ms/pixel.

### Antibunching Measurement

The photon correlation experiment was conducted on a WITec alpha 300 microscope using a 50× objective (LD EC “Epiplan-Neofluar,” NA = 0.55, Carl Zeiss Microscopy, LLC), and the excitation laser was a CW 532 nm laser. The incident laser intensity, exposure time, and accumulations were 5 mW, 1 s, and 300 accumulations, respectively. The emitted photons were filtered with a 580 nm long-pass filter and collected with avalanche photodiodes (APD, MPD PDM Series). The APD outputs were connected to a timing module (PicoHarp 150, PicoQuant), which recorded the arrival time of each photon.

### Fluorescence Lifetime Measurement

For the time-resolved photoluminescence experiments, a 405 nm picosecond laser (LDH-D-C-405, PicoQuant) was used as the excitation source. Laser excitation power, repetition rate, and integration time were 0.968 mW, 40 MHz, and 2 s, respectively. The emitted signal was filtered through a 580 nm long-pass filter and detected using an avalanche photodiode (MPD PDM Series). The APD output was connected to a timing module (PicoHarp 150, PicoQuant) to record the arrival time of photons.

### Nano-FTIR Measurement

A commercial nano-FTIR system (neaSCOPE, attocube systems AG) was used for near-field infrared spectroscopy to probe molecular vibrational signals. The PtIr coated AFM tip (EFM-50, Nano World) was operated in tapping mode to minimize background noise, with the tip oscillating at an amplitude of *A* = 90 nm and a frequency of Ω = 73 kHz, using demodulation orders *n* = 2 and 3. The amplitude and phase spectra of nano-FTIR were both normalized with those of a clean silicon substrate.

## Supplementary Material



## Notes

AFM information; hBN thickness dependent FTIR spectra; nano-FTIR optical path; photoluminescence excitation optical path and results; fluorescence lifetime (PDF)

## Abbreviations

hBN: hexagonal boron nitride
SPEs: single photon emitters
TMDCs: transition-metal dichalcogenides
TEM: transmission electron microscope
PL: photoluminescence
EDX: energy-dispersive X-ray spectroscopy
FTIR: Fourier transform infrared spectroscopy
PDMS: polydimethylsiloxane
PAHs: polycyclic aromatic hydrocarbons
