# Supplementary material for: Identifying the Origin of Hexagonal Boron Nitride Single Photon Emitters with nano-FTIR
Source: J Phys Chem Lett. 2026 Feb 11;17(8):2197–204. doi: 10.1021/acs.jpclett.5c03632 (PMC12951557; doi:10.1021/acs.jpclett.5c03632)
Supplement: Supplementary file 1 [file jz5c03632_si_001.pdf]

Supporting Information

Identifying the Origin of Hexagonal Boron Nitride Single Photon Emitters with nano-FTIR

Chia-Hung Wu[1,2], Po-Sheng Shih[3], Nicholaus Kevin Tanjaya[2,4], Kuo-Ping Chen[3,] and Satoshi Ishii[2,4,*]*

[1] College of Photonics, National Yang Ming Chiao Tung University, 301 Gaofa 3rd Road, Tainan 71150, Taiwan

[2] Research Center for Materials Nanoarchitectonics (MANA), National Institute for Materials Science (NIMS), 1-1 Namiki, Tsukuba, Ibaraki 305-0044, Japan

[3] Institute of Photonics Technologies, National Tsing Hua University, Hsinchu 300, Taiwan

[4] Subprogram in Materials Science and Engineering, Graduate School of Science and Technology, University of Tsukuba, Tsukuba, Ibaraki 305-8577, Japan

*sishii@nims.go.jp; kpchen@ee.nthu.edu.tw

KEYWORDS: Hexagonal boron nitride, Nano-FTIR, Single photon emitter, Photoluminescence, Organic molecule

Flake 1 AFM image:

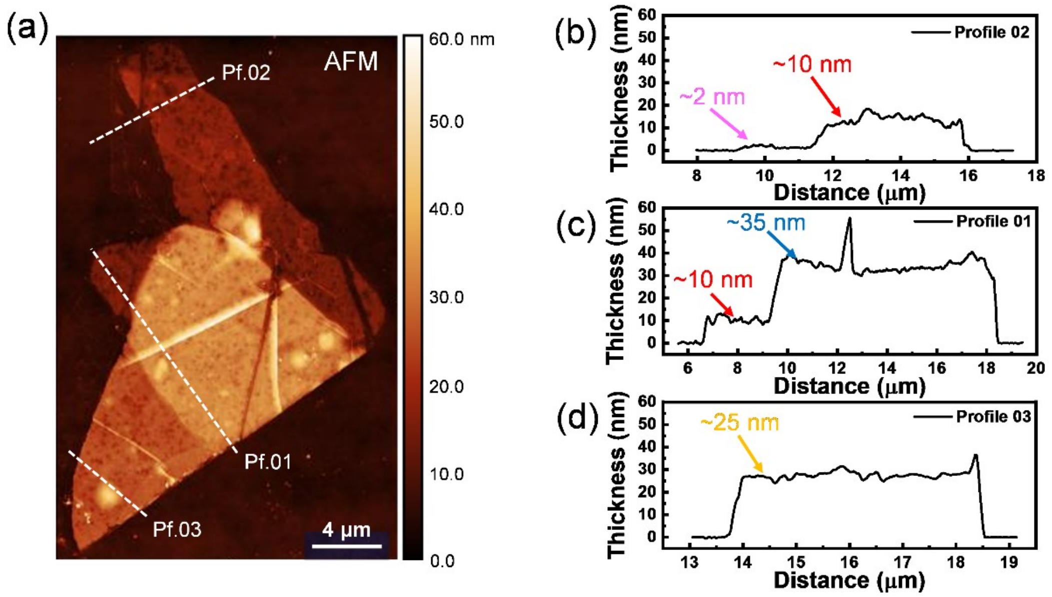

Figure S1 (a) AFM image for Flake 1. (b), (c), and (d) are thickness profiles of different areas of the flake. Based on the results, Flake 1 is composed of two partially stacked flakes.

ZPL and $g^{(2)}(0)$ dip variation:

Additional photoluminescence (PL) spectra and their $g^{(2)}(0)$ dips were presented for nine different SPEs. The PL features the ZPL around 610 nm. For the $g^{(2)}(0)$ dips, only three out of nine locations have reached the criteria below 0.5.

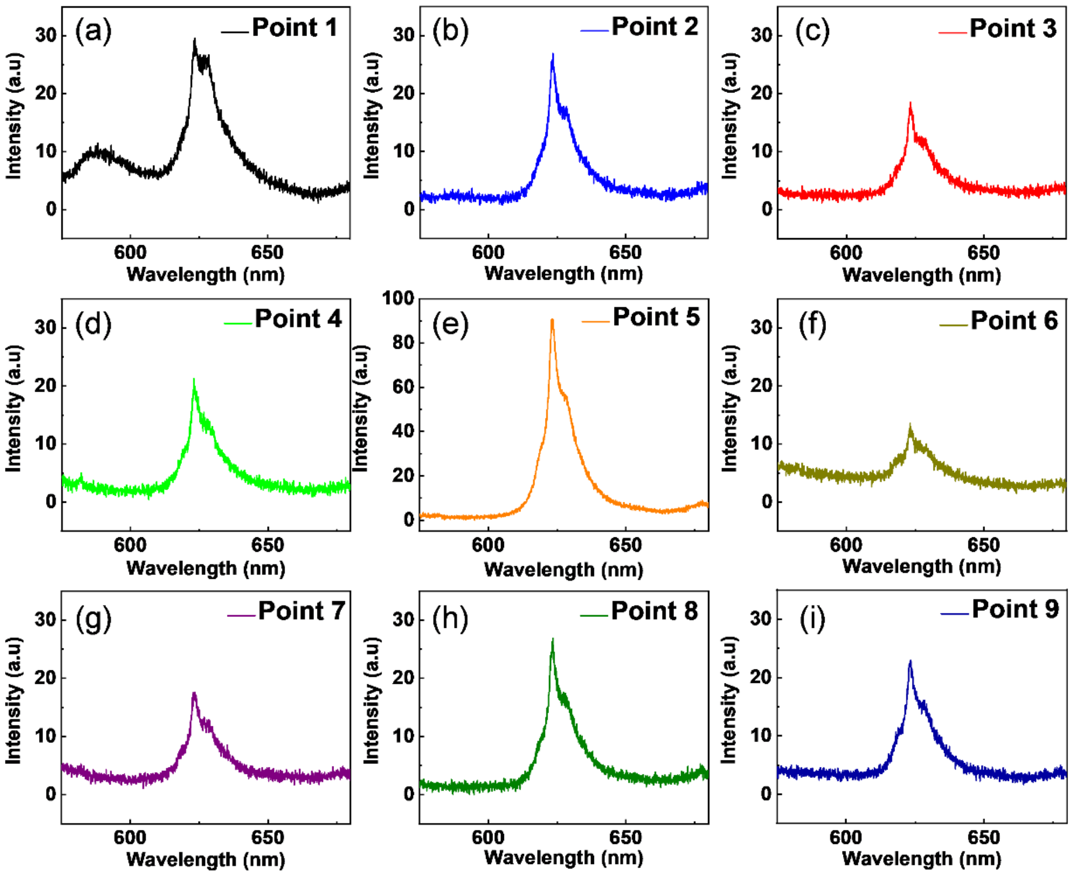

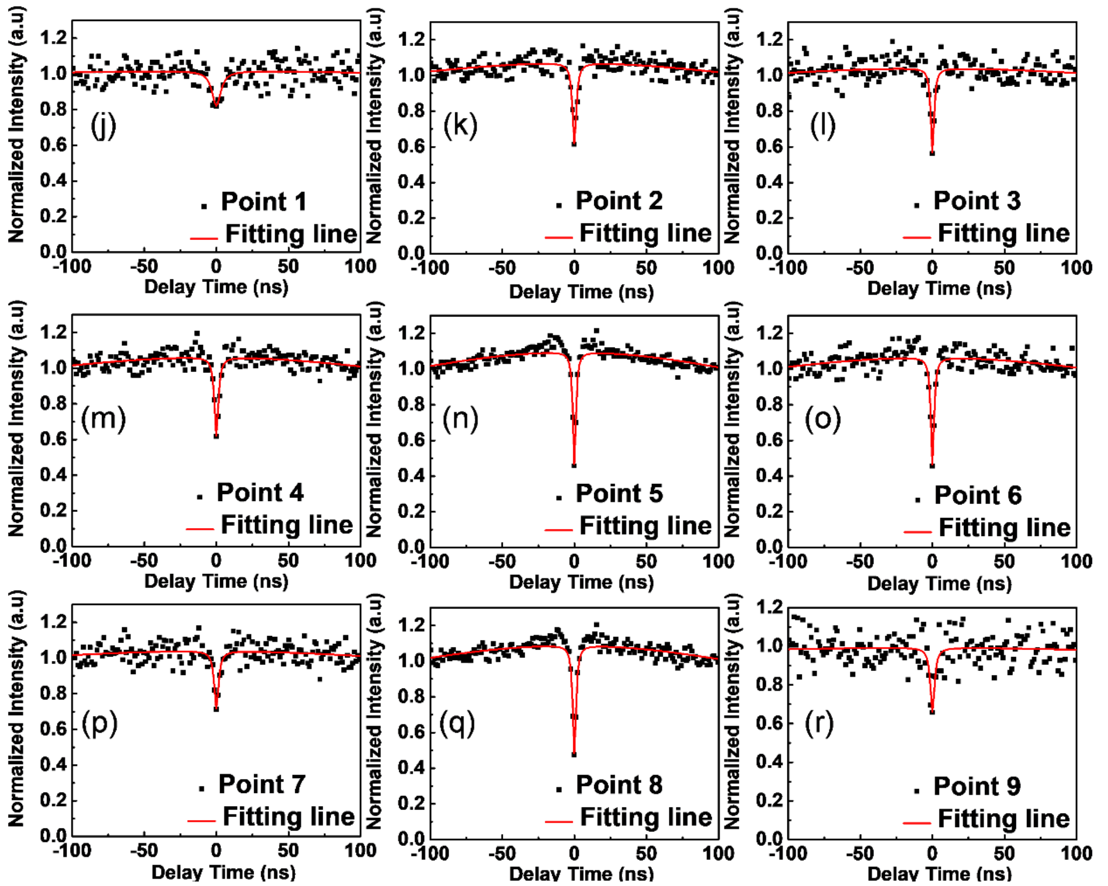

Figure S2. (a~i) Photoluminescent spectra and (j~r) $g^{(2)}(\tau)$ curves collected from nine different locations.

Blinking and saturation of emitter:

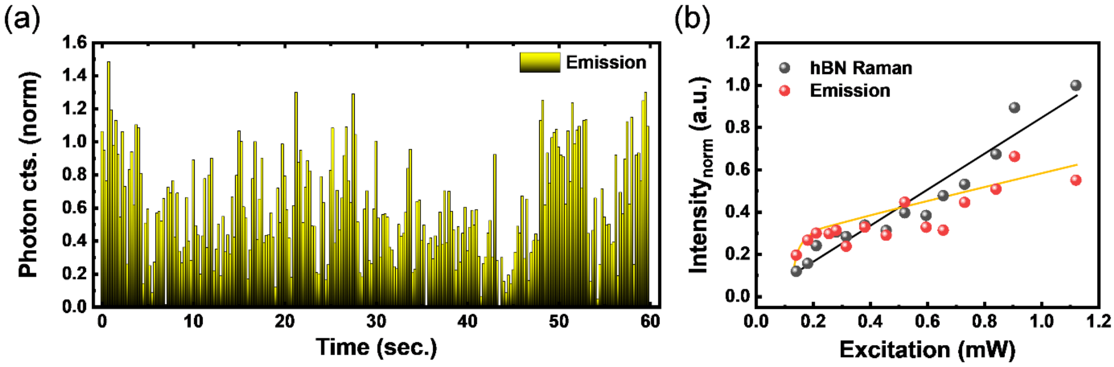

Figure S3. Photophysical properties of Type I specimen. (a) Time resolved photoluminescence intensity. (b) Power dependent fluorescence intensity. The spheres represent the measured data, whereas the black and orange lines represent the fitted hBN E_{2g} and emission signals, respectively. Emission wavelength of interest set at 610 nm.

Photoluminescence excitation (PLE):

Photoluminescence excitation (PLE) focuses on the interaction between electromagnetic radiation and matter. It is employed in spectroscopy to study how varying the excitation light frequency affects luminescence at the material's emission frequency. Peaks in PLE spectra generally represent the material's absorption lines. This concept could be extended further by varying the polarization of excitation, which provides the emissive specimen's sensitivity under different polarizations. **Figure S5** illustrates that luminescent sites with antibunching traits exhibit dipole-like polarization behavior in both emission and absorption. In contrast, luminescent sites without antibunching show consistent PL intensity regardless of polarization, pointing to clusters of single-photon emitters with diverse dipole orientations at that location.

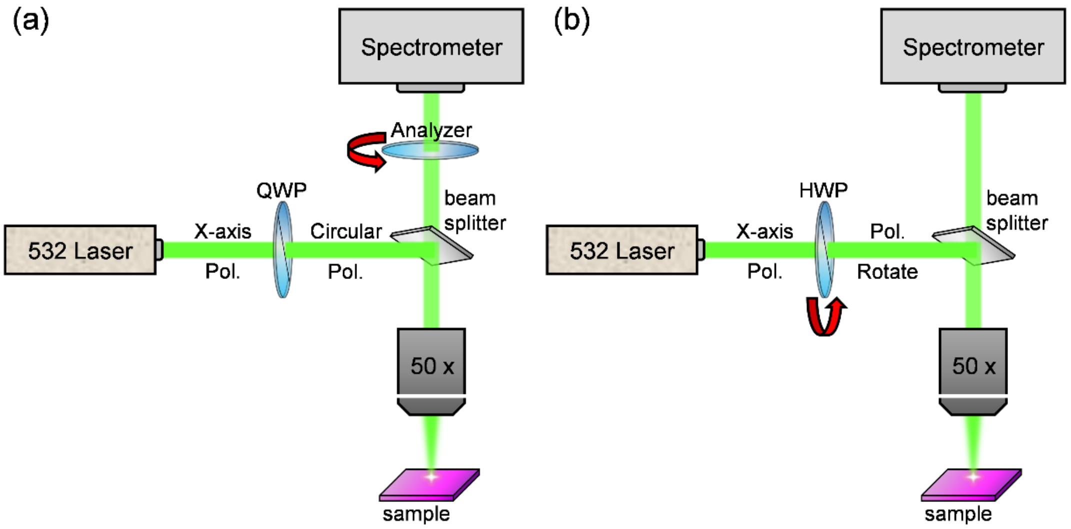

Figure S4. Optical setup of the PLE experiment. (a) The emission polarization was recorded with an analyzer before the spectrometer with a circularly polarized excitation. (b) The spectrometer recorded the absorption under excitations of different linear polarization angles.

Antibunching and SPE:

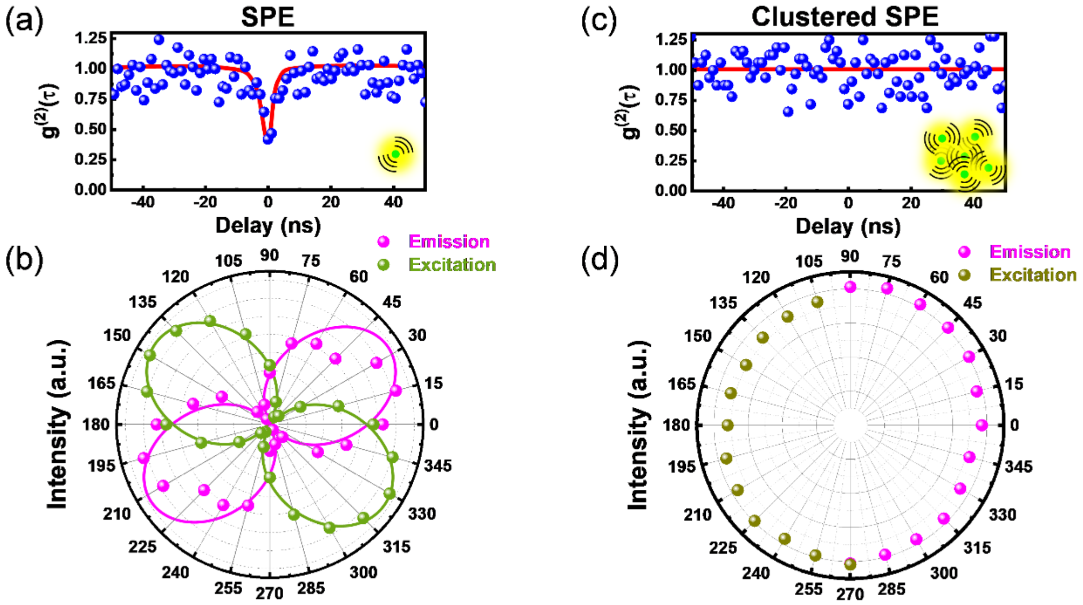

Figure S5. Comparison of PLE results with and without antibunching luminescent sites. (a) Second-order correlation function measurement on hBN with SPE traits. (b) PL intensity as a function of polarization angle for emission (purple data) and excitation (green data) with circular and linear polarized lasers, respectively. (c) Second-order correlation function measurement on hBN without SPE traits. (d) PL intensity as a function of polarization angle for emission (purple data) and excitation (green data) with circular and linear polarized lasers, respectively.

Polarization analysis:

The polarization states of three different emitters were analyzed. As the polarization directions are random, the SPEs do not align with the hBN crystal axes.

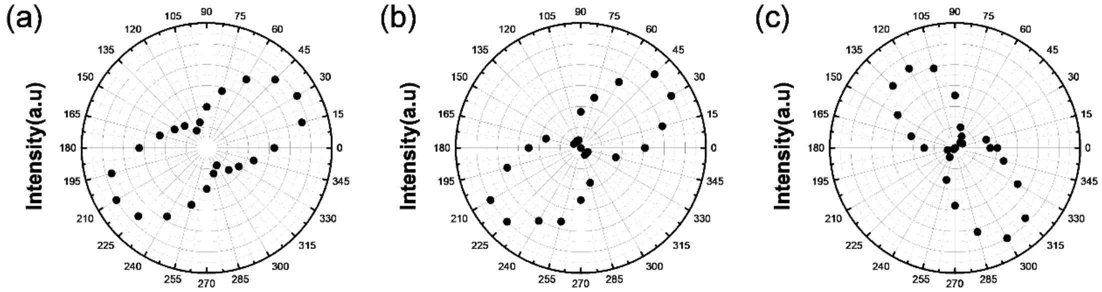

Figure S6. (a~c) Polarization-dependent emission spectra of three different SPEs.

Nano-FTIR optical path:

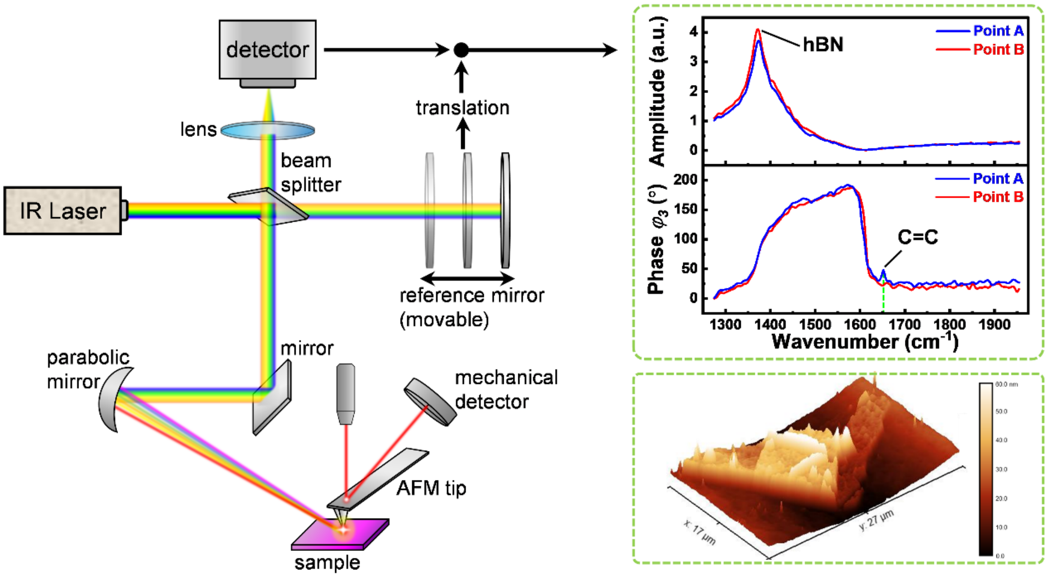

Figure S7. Optical setup of the nano-FTIR. Broadband IR-source was irradiated on the AFM tip apex creating a strong confinement, which acted as a nano-white light source that probed the sample. The interferogram was translated into spectra to obtain amplitude and phase.

Additional nanoFTIR spectra:

We performed nanoFTIR measurements at six different locations, which also showed the C=C double bond signals at 1650 cm^{-1}.

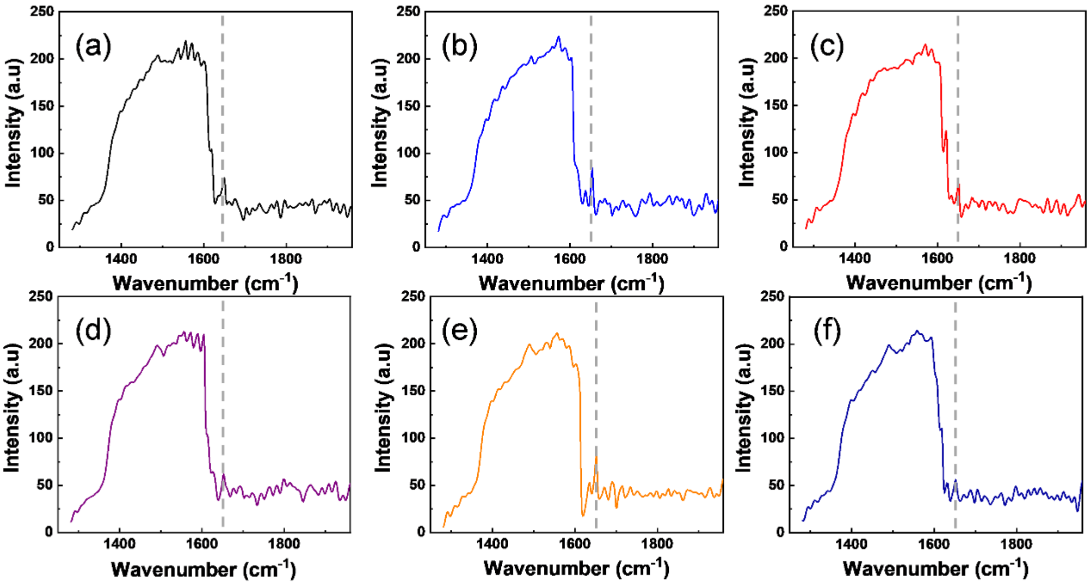

Figure S8. Nano-FTIR spectra of hBN measured at six different locations.

Flake thickness variation FTIR phase:

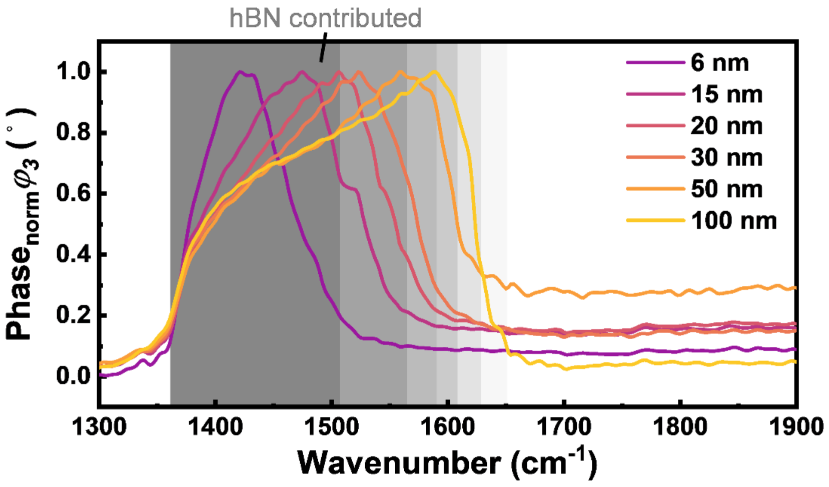

Figure S9. FTIR phase spectraa of hBN flakes with different thicknesses. The grey shaded areas indicate the hBN-contributed broadband peak. Note that this peak range is fully correlated to the hBN thickness.

Oxidative burning of graphite:

In this section, we investigate the same Type II flake as shown in the main text **Figure 5**. **Figure S10 (**a) shows the optical bright field image of the hBN flake, the inset is the mapping image for hBN Raman signal, indicative of hBN presence. Notably, spectra taken from both the hBN flake and substrate show identical fluorescence profiles as depicted in **Figure S10** (d), with wavelengths at 582 nm (carbon G-band) and a broad emission around 630 nm (carbon 2D-band), except for a sharp peak at 573 nm observed only on the flake, indicative of the hBN signature peak. The dark box observed in **Figure S10** (c) resulted from the photobleaching of the graphite on the sample surface during the first laser scan which is provided in **Figure S10** (b). The carbons protected by the hBN flake maintained their signal. The Raman signal intensity of hBN, presented as black circles in **Figure S10** (e), was provided to demonstrate the stability of the measurement system and confirm that no modifications were made to the hBN during the experiment. **Figure S10** (f) shows nano-FTIR spectra measured on the substrate

before and after photobleaching the emitters. Results showed a distinctive peak at 1650 cm^{-1} before bleaching, which disappeared after laser illumination, as anticipated.

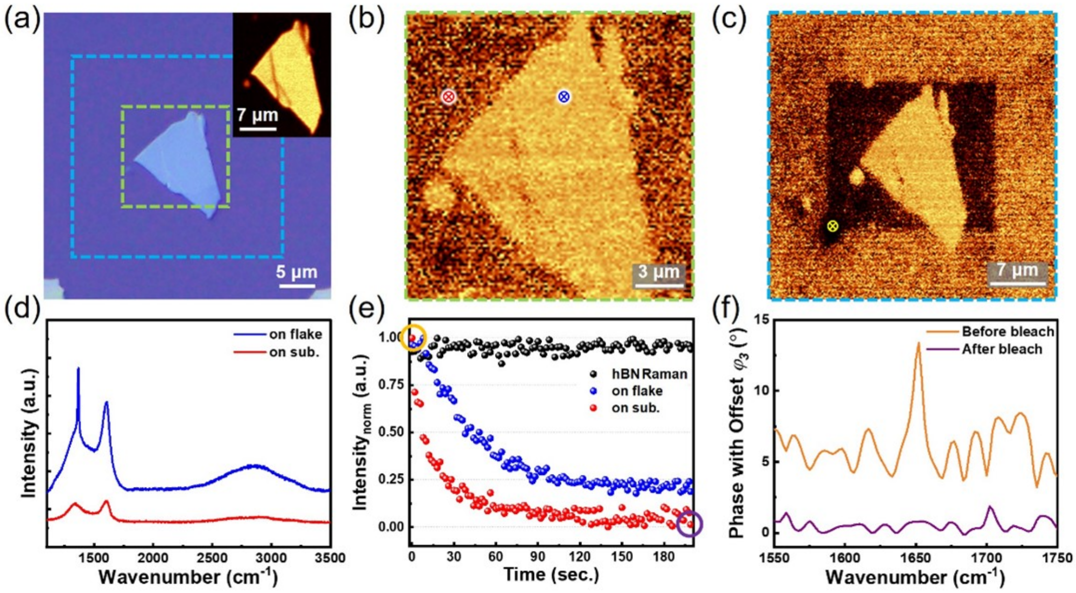

Figure S10 Demonstration of oxidative burning graphite on the substrate and hBN. (a) Optical microscope image of a Type II specimen. The green and cyan dashed boxes illustrate the areas of 1st and 2nd laser scanning region, respectively. Inset: Mapping of hBN signature Raman peak. (b) First laser scan mapping of 582 nm. The substrate area was also covered with graphite. The blue and red circles mark the measured points displayed in (d) and (e). (c) Second laser scan mapping of 582 nm. A “dark” region is present, showcasing the photobleaching caused by the first laser scan. The green circle marks the measured point displayed in (f). (d) Spectrum collected on the hBN and the substrate with 532 nm CW laser excitation. (e) Time-dependent carbon G-band signal intensity recorded on the hBN and substrate. (f) Third-order phase spectra φ_3 obtained on the substrate with nano-FTIR before and after photobleaching. The measured time was labeled as orange and purple circles in (e).

Fluorescence lifetime:

In this section, we investigate the photon lifetime of specimens with varying thicknesses using time-resolved photoluminescence (TRPL) experiments. As shown in the Table S1, the results indicate that the photon lifetime is independent of the thickness of hBN, with several distinct lifetime values observed. This suggests the presence of different aromatic fluorophores, implying the existence of multiple types of emitters.

Table S1. Fluorescence lifetime of emissive hBN specimens with thickness variations.

hBN thickness (nm)	τ (ns)
6	1.69 ± 0.08
15	3.03 ± 0.50
15	2.39 ± 0.24
20	3.31 ± 0.46
20	3.72 ± 0.82
30	3.46 ± 0.63
40	2.89 ± 0.35
55	2.15 ± 0.17
60	1.93 ± 0.13
100	2.69 ± 0.31
